# WEARCON: wearable home monitoring in children with asthma reveals a strong association with hospital based assessment of asthma control

**DOI:** 10.1186/s12911-020-01210-1

**Published:** 2020-08-14

**Authors:** M. R. van der Kamp, E. C. Klaver, B. J. Thio, J. M. M. Driessen, F. H. C. de Jongh, M. Tabak, J. van der Palen, H. J. Hermens

**Affiliations:** 1 Department of Paediatrics, Medisch Spectrum Twente, Enschede, Netherlands; 2 Department of Biomedical Signals and Systems, University of Twente, Enschede, Netherlands; 3 Roessingh Research and Development, Enschede, the Netherlands; 4 Medical School Twente, Medisch Spectrum Twente, Enschede, Netherlands; 5 OCON sport, Hengelo, Netherlands; 6 Department of Sports Medicine, Ziekenhuis Tjongerschans, Heerenveen, Netherlands; 7 Department of Pulmonology, Medisch Spectrum Twente, Enschede, Netherlands; 8 Department of Engineering Fluid Dynamics, University of Twente, Enschede, Netherlands; 9 Department of Research Methodology, Measurement and Data Analysis, University of Twente, Enschede, Netherlands

**Keywords:** Asthma control, Ambulatory monitoring, eHealth, Physiology sensors, Wearable electronic devices, Paediatrics, Telemedicine, Multivariate analysis, spirometry, Inhaler use

## Abstract

**Background:**

Asthma is one of the most common chronic diseases in childhood. Regular follow-up of physiological parameters in the home setting, in relation to asthma symptoms, can provide complementary quantitative insights into the dynamics of the asthma status. Despite considerable interest in asthma home-monitoring in children, there is a paucity of scientific evidence, especially on multi-parameter monitoring approaches. Therefore, the aim of this study is to investigate whether asthma control can be accurately assessed in the home situation by combining parameters from respiratory physiology sensors.

**Methods:**

Sixty asthmatic and thirty non-asthmatic children were enrolled in the observational WEARCON-study. Asthma control was assessed according to GINA guidelines by the paediatrician. All children were also evaluated during a 2-week home-monitoring period with wearable devices; a physical activity tracker, a handheld spirometer, smart inhalers, and an ambulatory electrocardiography device to monitor heart and respiratory rate. Multiple logistic regression analysis was used to determine which diagnostic measures were associated with asthma control.

**Results:**

24 of the 27 uncontrolled asthmatic children and 29 of the 32 controlled asthmatic children could be accurately identified with this model. The final model showed that a larger variation in pre-exercise lung function (OR = 1.34 95%-CI 1.07–1.68), an earlier wake-up-time (OR = 1.05 95%-CI 1.01–1.10), more reliever use (OR = 1.11 95%-CI 1.03–1.19) and a longer respiratory rate recovery time (OR = 1.12 95%-CI 1.05–1.20) were significant contributors to the probability of having uncontrolled asthma.

**Conclusions:**

Home-monitoring of physiological parameters correlates with paediatrician assessed asthma control. The constructed multivariate model identifies 88.9% of all uncontrolled asthmatic children, indicating a high potential for monitoring of asthma control. This may allow healthcare professionals to assess asthma control at home.

**Trial registration:**

Netherlands Trail Register, NL6087. Registered 14 February 2017.

## Background

Asthma is one of the most common chronic diseases in childhood and has a major impact on the quality of life [1, 2]. Paediatric asthma is characterized by chronic airway inflammation and bronchial hyperresponsiveness to triggers such as allergens, exercise and viral infections. Symptoms include shortness of breath, wheeze and cough hampering sleep, play and sports [3]. National and international respiratory associations recognize the scale and impact of this chronic lung disease [4, 5].

The Dutch lung alliance states that regular follow-up of asthma control is needed to prevent disease deterioration and boost quality of life [4]. However, scheduled outpatient-clinic evaluations at infrequent intervals do not always follow the fluctuating course of paediatric asthma symptoms. Moreover, this follow-up normally requires extensive evaluation in a hospital setting to accurately assess the asthma status of a child according to the guidelines of the Global Initiative for Asthma (GINA) (i.e. the assessment of asthma symptom control, monitoring risk factors (lung function, airway hyperresponsiveness and exacerbations) and assessing treatment factors (adherence/inhalation technique)) [5]. Ambulant monitoring provides opportunities to objectively follow-up physiological parameters by longitudinal measurements in daily life, outside regular visits, and may provide healthcare professionals with complementary insights into the dynamics of the asthma status.

Asthma control questionnaires are used to assist in monitoring symptom severity in the home-situation [6–8]. These questionnaires offer an easy low-cost option to follow-up symptom control on a regular basis. However, they are also prone to symptom misperception, individual interpretation of the questions, and recall bias [9, 10]. Moreover, children quickly adapt their behaviour to pathophysiological decline in asthma control and consequently report no or subtle symptoms, while the decline might be serious [11, 12]. Monitoring the questionnaire scores alone has yet not been able to improve symptom management or impact daily life [13]. This stresses the urge for additional complementary objective methods to monitor children with asthma at home, providing real-time assessment of symptoms and physiological modulation [14].

The most frequently investigated home-monitoring device dates back from the pre-technology-era and is the peak expiratory flow meter [15]. Kotses et al. [16] concluded that peak flow only gives a small increment in effectiveness beyond that afforded by symptom monitoring. In the last decade, literature also reveals increasing efforts in monitoring medication adherence at home to steer asthma management [17]. Other home-monitoring studies involved measurements of physical activity [18, 19], inflammation markers [20], respiratory distress [21]^,^ or coughing and wheezing [22]. All individual parameters showed potential in monitoring asthma but were individually not strongly related to control of asthma in a broad paediatric population.

Despite considerable interest in asthma home-monitoring in children, there is a paucity of scientific evidence, especially on multi-parameter monitoring approaches [13, 23]. We hypothesize that a holistic home-monitoring approach, combining the outcomes of multiple wearable devices signalling respiratory physiology, can provide quantitative relevant information on paediatric asthma control. Therefore, the objective of the WEARCON study is to investigate whether asthma control can be accurately assessed in the home-situation with a combination of measurements from respiratory physiology sensors.

## Methods

### Study design

**Fig. 1:**
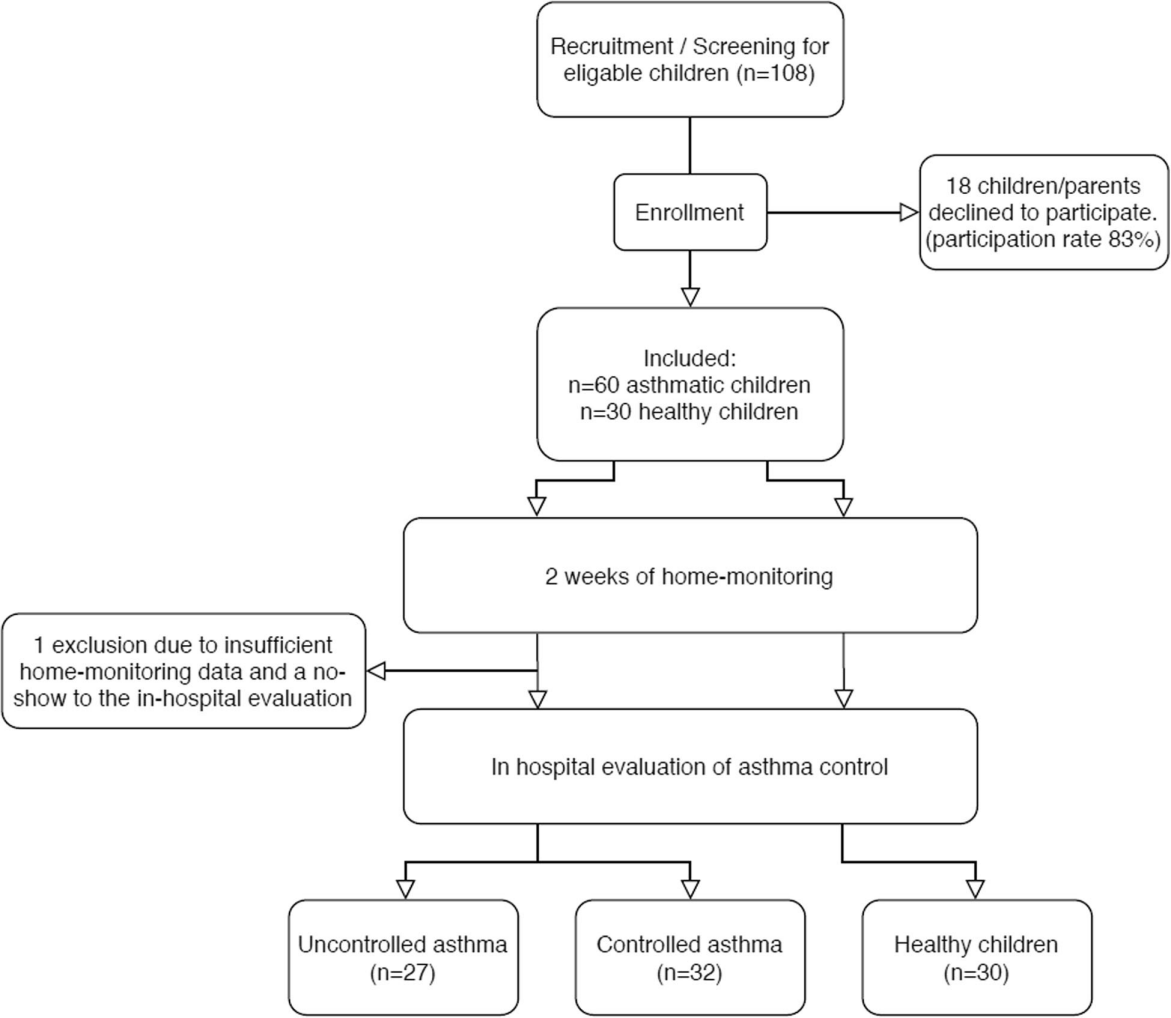
Schematic overview of the study design. Legend: Schematic overview of the study design; describing the process of recruitment, enrolment, home-monitoring, and grouping based on the outpatient-clinic evaluation

The WEARCON study had a prospective observational design. After informed consent, children and parents received all study devices, instruction materials, and were instructed at their home. Children were monitored for 2 weeks at home with wearable devices, followed by an outpatient-clinic visit to assess asthma control (Fig. 1). This study was approved by the medical ethics committee and was registered in the Netherlands trial register (trial no. NL6087). Oral and written consent to participate were obtained from the parents or legal guardians of the children. Children of 12 years or older also provided oral and written consent.

### Subjects

Sixty children with paediatrician-diagnosed asthma and thirty non-asthmatic children between 4 and 14 years, were recruited using consecutive sampling.

#### Asthmatic children (*n* = 60)

The asthmatic children were recruited at the outpatient clinic of the paediatric department of Medisch Spectrum Twente, Enschede, The Netherlands (referral centre for paediatric asthma). Children with paediatrician-diagnosed asthma, who had exercise induced symptoms and were scheduled for an exercise bronchoprovocation test (BPT) between February 2017 and June 2018, were approached to participate in the study. Children with comorbid chronic diseases, children with an inability to understand or speak Dutch, children with electrical stimulation devices (i.e. pacemaker), children with psychomotor retardation, or children for whom it was not possible to wear all wearable devices, i.e. due to severe skin diseases or amputation, were not eligible to participate.

Asthma control was assessed in every child by the same paediatric pulmonologist according to the GINA recommendations of assessment of asthma control [5]. Many children with poorly controlled asthma avoid strenuous exercise or mispercept symptoms, so their asthma may appear to be well controlled [5]. Therefore, the BPT was used in addition to the GINA recommendations to assess asthma control. Uncontrolled asthma was defined as 1) having an uncontrolled level of asthma symptom control as defined by GINA (three or more of the following conditions in the past 4 weeks; > 2 episodes of daytime symptoms weekly, > 2 uses of reliever medication weekly, nocturnal symptoms and activity limitation) OR 2) having a positive BPT (> 12% decrease in FEV_1_) [5]. The exercise BPT was performed in a climate chamber with dry, cold air (10 degrees Celsius) following the American Thoracic Society protocol [24]. Children aged 8–14 years old performed the BPT on a treadmill for 6 min with submaximal exercise load (steady-state heart rate of 85% of the maximal heart rate (220 – age)) and their nose clipped. The inclination of the treadmill was 10%. Children aged 4–7 years old performed the exercise on a jumping castle for 6 min as described by van Leeuwen et al. [25].

#### Non-asthmatic children (*n* = 30)

The non-asthmatic controls were recruited with information flyers at schools in the region. The non-asthmatic children received the same medical evaluation to confirm that they did not have asthma. The same exclusion criteria applied for the non-asthmatic group. Children with a prior diagnosis of asthma, prescribed asthma medication or self-reported asthmatic symptoms, were ineligible.

### Subject characteristics

Demographic characteristics were retrieved from the electronic patient record. The (C)-ACT score was obtained after each week of monitoring. Lung function (FEV_1_% predicted) and the maximal post-exercise fall in FEV_1_ were obtained during the BPT.

### (Wearable) monitoring devices

**Fig. 2:**
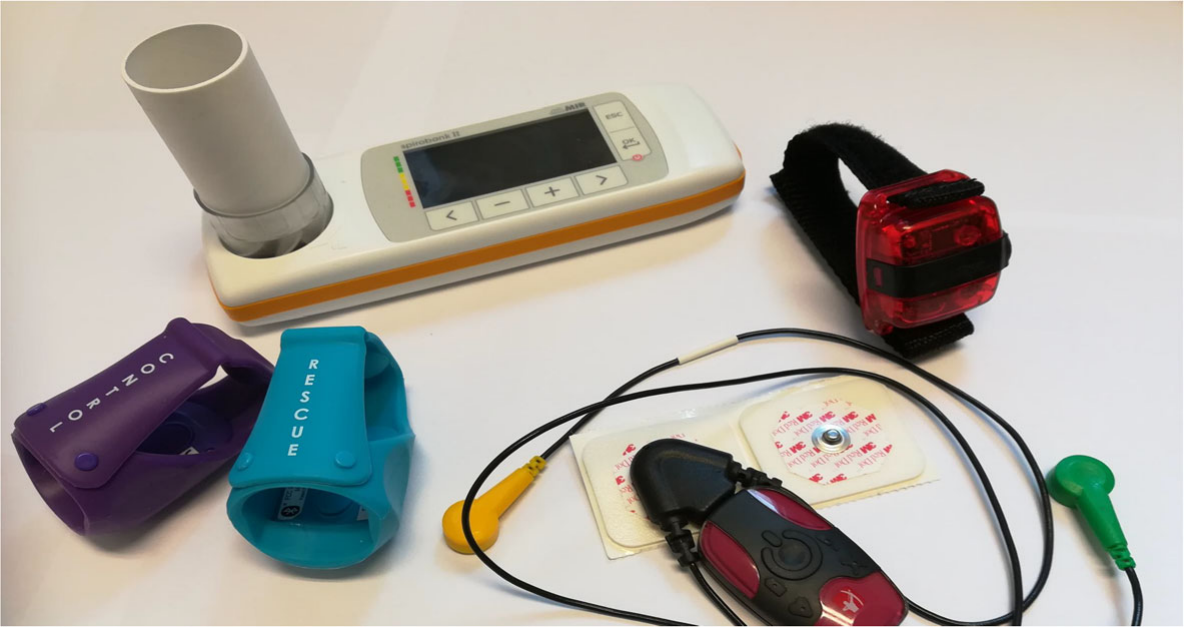
The smart monitoring devices. Legend: Smart devices from top-left to bottom-right: MIR spirobank II advanced, Actigraph wGT3X-BT, Cohero Health smart inhalers, eMotion Faros 180

Figure 2 shows the four commercially available devices used in the WEARCON study. Our choice of devices was based on the trade-off between 1) the best quality devices (so that the most relevant data could be extracted for this study) and 2) the non-obtrusiveness of the devices (so that it would be feasible for children to be able to use the device for 2 weeks). Physical activity was assessed using the Actigraph WGT3X-BT wireless activity tracker (Actigraph inc. Pensacola, FL). Lung function measurements were performed with the hand-held Spirobank advanced II (MIR inc. Roma, Italy). Medication adherence and reliever medication use were electronically tracked with the two Cohero Health smart inhalers. (Cohero inc. New York, NY). Electrocardiography (ECG) was measured using the Emotion Faros 180° (Bittium. Oulu, Findland). Wearables did not show interpretable data to the subjects to prevent any influence and data was stored anonymously.

### Data acquisition, preprocessing and analysis

Continuously measured signals had to be at least 75% complete to be eligible for pre-processing and analysis.

#### Physical activity & sleep

The subjects wore the activity tracker for fourteen consecutive days in representative school weeks, without (bank) holidays, reflecting the subjects’ average habitual activities [26]. The subjects were instructed to attach the tracker at the wrist and remove it only before activities involving water (such as showering or swimming). Physical activity outcome measures yielded the number of minutes spent at each of four activity levels (sedentary, light, moderate and vigorous activity), the average duration (bout length) and the distribution of activities from at least moderate intensity, expressed in the scale parameter of the Weibull distribution [27]. Sleep parameters were derived from the activity tracker with the Cole-Kripke sleep algorithm [28]. This algorithm provided the average sleep time, wake-up-time (defined in minutes after midnight), sleep efficiency, awake minutes and time per awakening. Furthermore, the sleep restlessness 1 h before wake-up was defined as the average vector magnitude activity counts in the hour the children wake-up. All activity and sleep parameters were averaged per day over the 2 weeks of home-measurement.

#### Spirometry measurements at home

Children were instructed to perform spirometry whenever they exercised (before and 3–6 min after) and during symptoms (before reliever use). Spirometer flow-volume loops were classified accordingly based on self-reported events (pre-exercise, post-exercise, symptom). Incorrectly blown spirometer measurements were excluded, according to the ATS/ERS criteria for standardisation lung function testing [29]. Spirometry outcome measures were the average pre-exercise forced expiratory volume in 1 s (FEV_1_), pre-exercise forced expiratory flow between 25 and 75% of exhalation (FEF_25–75_), pre-exercise peak expiratory flow (PEF), the percentage change in FEV_1_ after exercise and during symptoms and the variation in pre-exercise lung function, defined as the absolute difference between the highest and lowest predicted pre-exercise FEV_1_.

#### Smart inhaler

The date and time of inhalation were acquired from the Cohero Health server. Controller adherence was calculated by dividing the amount of controller medication taken by the amount of medication prescribed (%). Reliever usage was summed for the period of 2 week monitoring.

#### Heart rate and respiratory rate

Continuous raw ECG data was acquired for 2 days and two nights, with at least one vigorous activity within the period (sports, gym class). Subjects were instructed to attach the eMotion Faros device according to the 3-wire lead placement (mid-clavicular under both claviculae and on left abdomen within the rib cage frame). The device was removed before activities involving water.

The raw ECG was pre-processed to retrieve heart rate (HR) and respiratory rate (RR) using ECG-derived respiration, which is known to provide an robust RR estimate [30]. Artefact and baseline correction was applied using a FIR filter with a Kaiser window using cut-off frequencies of 0.45 and 39 Hz [31]. The RS amplitude was determined by subtracting the S-amplitude from the R-amplitude of the same QRS complex. The respiratory curve based on the RS-amplitude was established by using cubic spline interpolation to construct a respiratory signal with 50 Hz [32]. This algorithm was validated against flow measurement on a separate set of subjects during different daily tasks, showing strong positive correlations (*r* = 0.69) and a sensitivity of 91.5% and positive predictive value of 0.998 on assessing single breathing cycles [33].

ECG outcome parameters were the average daytime HR and RR, night-time HR and RR (in beats or breath per minute) and the HR and RR recovery time, defined as the time (seconds) needed to recover to baseline after physical exertion.

### Statistical analysis

Descriptive statistics were used to examine all continuous outcome measures and were expressed in means + − standard deviation (SD) for normally distributed variables and with median + − interquartile range (IQR) for non-normal distributed variables. Univariate analyses were performed with SPSS statistics (IBM Corp. Released 2013, Version 22.0). The differences in the categorical variables across the different asthma groups were tested with a chi-square test. Homogeneity of variances was verified in all continuous outcome parameters with the Levene’s test. The Shapiro-Wilk test was used to determine whether the variables were normally distributed among all three groups. The differences across the asthma groups in the variables that did not have a normal distribution were tested with the Kruskal-Wallis test followed by multiple comparisons of Games-Howell. The difference of normally distributed variables across the asthma groups were tested with Analysis of Variance (ANOVA) followed by Tukey HSD test for the post-hoc comparisons of the three groups. *P*-values less than 0.05 were considered as significant.

Prior to the multivariate analysis missing data was handled using the multiple imputation regression method. Missing data patterns were analysed for monotonicity. In case of monotonicity the monotone method was used; in case of random patterns the Markov Chain Monte Carlo method was used. Constraints were added to the variables to prevent unrealistic imputations (e.g. negative lung function values). Five imputed datasets were created and pooled according to the bar procedure [34]. Multivariate analysis was performed using a binary logistic regression analysis with asthma control as dependent variable, with the controlled asthma group as reference group, as the intended use of the model is to assist in the monitoring of children who are already diagnosed with asthma. All home monitoring parameters (see Table 2) were considered for inclusion in this final multivariate model. Independent variables with a multi-collinearity of more than 0.8 were not both used in the same model. The model was not adjusted for other potential predictors, such as age, gender, allergies etc., to prevent overfitting of the model and to specifically focus the model on the best combination of home-monitoring parameters. Stepwise forward likelihood ratio selection was used as enter method of variables with an entry probability of 0.10 and removal probability of 0.20. The model was optimized using the Nagelkerke pseudo R-squared, so that the model which explained the most of the variation (R^2^ closest to 1.0) was chosen. The resulting binary logistic regression was used to determine relevant diagnostic validity measures, such as sensitivity, specificity and positive and negative predictive value.

### Sample size

WEARCON studied whether asthma control could be accurately assessed using a multiple binary logistic regression model. Agresti and Peduzzi suggested ten cases per event per group [35, 36]. This indicated that for a three parameter multiple regression model 60 (30/30) asthmatic children were needed, assuming an equal distribution between the children with controlled and uncontrolled asthma [37]. Thirty non-asthmatic children were included as well to put all asthma home-monitoring parameters in perspective relative to normal values and opens the opportunity to explore the diagnostic value of these parameters for asthma in general.

## Results

The participation rate of all eligible children for this study was 83.3% (90/108). From these 90 subjects, one was excluded due to insufficient home-monitoring data and a no-show to the outpatient-clinic evaluation. Overall data completeness was 88.5%. On average, children performed nine spirometry measurements over the course of 2 weeks. 73,9% of these attempts were satisfactory according to the ATS/ERS criteria for standardisation lung function testing. The wear time of the activity tracker was 91,7% (±SD 9,9%) during daytime. The sleep data was complete for 94,4% of the nights.

### Asthma control classification

Of the remaining 89 children, thirty-two were placed in the “controlled asthma group”, twenty-seven in the “uncontrolled asthma group” and thirty children were included as non-asthmatic subjects. From the twenty-seven uncontrolled asthmatics, thirteen were classified uncontrolled based on the results of the BPT, three on the GINA criteria and eleven on both the GINA and BPT results. Table 1 shows an overview of the subject characteristics of all children. Significant baseline differences in the presence of allergy and maximal FEV_1_ fall at the BPT were found between the children with controlled asthma compared to the children with uncontrolled asthma.

**Table 1:** Subject characteristics. Data are shown as mean ± SD, %, or median (IQR)

	Uncontrolled asthma (***n*** = 27)	Controlled asthma (***n*** = 32)	Non-asthmatics (***n*** = 30)	***P***-value (ANOVA / Kruskal-Wallis / Chi-square)
**Age** (y)	8.2 ± 2.8	9.5 ± 2.6	9.3 ± 2.9	0.19 *
**Gender** (% male)	77%	84%	53% ^a^	0.02 +
**Weight** (kg)	31.4 ± 10.6	37.9 ± 14.2	32.9 ± 11.5	0.11 *
**Length** (cm)	133 ± 17	140 ± 17	138 ± 18	0.30 *
**BMI z-score**	0.43 ± 0.94	0.85 ± 1.17	−0.02 ± 1.13 ^a^	< 0.01 *
**ICS use** (%)	70%	66%	0% ^c,d^	< 0.01 +
**LABA use** (%)	11.1%	6.7%	0% ^c,d^	0.19 +
**Inhalation allergy** (%)	90%	61% ^b^	13% ^c,d^	< 0.01 +
**Baseline FEV**_**1**_ (% pred)	90.6% ± 12.7%	92.9% ± 9.8%	95.7% ± 9.7%	0.21 *
**Fall in FEV**_**1**_ **at ECT** (%)	27.9% (17.3–32.8%)	6.0% (3.8–9.8%) ^d^	3.1% (0.6–5.1%) ^d^	< 0.01 ^
**C-ACT scores**	22.0 (17–25)	22.5 (20–25.5)	27.0 (27–27) ^c,d^	< 0.01 ^

Abbreviations: *BMI* Body mass index, *ICS* Inhaled corticosteroids, *LABA* Long acting beta-antagonists, *FEV*_*1*_ Forced expiratory volume in 1 s, *ECT* Exercise challenge test, *C-ACT* Childhood asthma control test

^a^*P* ≤ 0.05 compared to controlled asthma

^b^*P* ≤ 0.05 compared to uncontrolled asthma

^c^*P* < 0.01 compared to controlled asthma

^d^*P* < 0.01 compared to uncontrolled asthma

*Normally distributed (ANOVA)

^ Not normally distributed and/or inhomogeneity of variances (Kruskal-Wallis)

+ Binary variables > Chi-square

### Univariate analysis

Table 2 shows the univariate analysis of the home-measured parameters in each study group. The spirometry parameters (Table 2.a) showed that both the children with controlled and uncontrolled asthma had significantly lower home-measured pre-exercise lung function values (FEV_1_, FEF_25–75_ and PEF) compared the non-asthmatic controls. The children with uncontrolled asthma showed a significant larger variation in pre-exercise FEV_1_ and a larger FEV_1_ decrease after exercise and during symptoms compared to the controlled asthma group. No activity parameters showed any significant differences between the controlled, uncontrolled and non-asthmatic group (Table 2.b). Regarding sleep (Table 2.c), no significant differences were found in total sleep time and sleep efficiency. However, children with uncontrolled asthma woke-up earlier compared to children with controlled asthma. The uncontrolled asthma group furthermore showed on average a longer duration per awakening and more sleep restlessness in the hour before wake-up compared to the non-asthmatic children. Reliever use was significantly higher in the uncontrolled asthma group compared to the controlled asthma group (Table 2.d). The mean respiratory rate during night was higher in the uncontrolled asthma group compared to both the controlled and non-asthmatic group. The recovery time of both the heart rate and respiratory rate after exercise were higher in the uncontrolled asthma as well (Table 2.e).

**Table 2:** Univariate analysis of home-monitoring parameters in the domains; (a) Spirometry, (b) activity, (c) sleep, (d) medication use, (e) heart rate and respiratory rate. Data are shown as mean ± SD or median (IQR)

a) ***Spirometry***	**Uncontrolled**	**Controlled**	**Non-asthmatic**	***P*-value** **(ANOVA / Kruskal-Wallis)**
**Pre-exercise FEV**_**1**_ (% predicted)	82.2 ± 16.0	86.1 ± 8.9	98.0 ± 9.5 ^a,b^	< 0.01 *
**Pre-exercise FEF**_**25–75**_ (% predicted)	66.9 ± 20.9	75.3 ± 17.7	90.0 ± 20.5 ^a,b^	< 0.01 *
**Pre-exercise PEF** (% predicted)	77.4 ± 22.2	81.7 ± 14.4	98.1 ± 18.2 ^a,b^	< 0.01 *
**Variation pre-exercise FEV**_**1**_ (% predicted)	18.0 ± 10.3	9.4 ± 5.4 ^b^	7.6 ± 4.3 ^b^	< 0.01 *
**FEV**_**1**_ **change after exercise** (%)	− 11.5 ± 11.9	−0.6 ± 7.6 ^b^	−1.6 ± 3.7 ^b^	< 0.01 *
**FEV**_**1**_ **change during symptoms** (%)	−30.2 ± 21.4	−6.1 ± 8.1 ^b^	–	< 0.01 *
b) ***Activity***	**Uncontrolled**	**Controlled**	**Non-asthmatic**	***P*-value** **(ANOVA / Kruskal-Wallis)**
**Sedentary activity** (min/day)	568 ± 97	566 ± 90	573 ± 89	0.97 *
**Light activity** (min/day)	270 ± 50	274 ± 51	270 ± 47	0.92 *
**Moderate activity** (min/day)	97 ± 35	88 ± 33	92 ± 30	0.65 *
**Vigorous activity** (min/day)	7.4 ± 8.5	6.0 ± 7.9	9.4 ± 9.2	0.95 *
**Activity length** (seconds)	22.7 ± 2.3	21.9 ± 2.4	22.5 ± 2.3	0.39 *
**Scale parameter** (a.u.)	12.9 ± 1.9	12.2 ± 1.9	12.7 ± 1.8	0.33 *
c) ***Sleep***	**Uncontrolled**	**Controlled**	**Non-asthmatic**	***P*-value** **(ANOVA / Kruskal-Wallis)**
**Wake-up-time** (h:min)	6:28 (6:17–6:59)	7:18 (7:00–7:34) ^b^	7:01 (6:45–7:20)	< 0.01 ^
**Awake minutes per night** (min)	59.9 ± 19.9	51.6 ± 16.5	53.1 ± 17.5	0.19 *
**Time per awakening** (min)	2.66 ± 0.82	2.40 ± 0.87	2.36 ± 0.53 ^b^	0.06 *
**Sleep efficiency** (%)	89.0 ± 3.8	91.2 ± 5.0	90.4 ± 3.0	0.23 *
**Total sleep time per night** (min)	496 ± 62	514 ± 81	498 ± 54	0.60 *
**Sleep restlessness before wake-up** (counts)	3.37 (2.56–4.87)	2.79 (1.91–4.12)	2.76 (2.48–3.18) ^b^	0.07 ^
d) ***Medication use***	**Uncontrolled**	**Controlled**	**Non-asthmatic**	***P*-value** **(ANOVA / Kruskal-Wallis)**
**Reliever use** (n.o.u.)	16.5 (1–34)	3 (0–5) ^b^	–	0.04 ^
**Reliever use after activity** (n.o.u.)	0.5 (0–5.5)	0 (0–0) ^b^	–	< 0.01 ^
**Reliever use before activity** (n.o.u.)	0 (0–5)	0 (0–0) ^b^	–	< 0.01 ^
**Controller adherence** (% of prescribed)	81.1 ± 30.9	92.7 ± 19.1	–	0.24 *
e) ***Heart rate & respiratory rate***	**Uncontrolled**	**Controlled**	**Non-asthmatic**	***P*-value** **(ANOVA / Kruskal-Wallis)**
**Daytime heartrate** (beats/min)	101 ± 17	97 ± 16	102 ± 10	0.08 *
**Daytime respiratory rate (breaths/min)**	19.7 ± 2.9	18.5 ± 2.2	19.3 ± 2.0	0.16 *
**Nighttime heartrate** (beats/min)	79 ± 16	72 ± 14	71 ± 9	0.36 *
**Nighttime respiratory rate** (breaths/min)	17.5 ± 2.6	15.6 ± 1.6 ^b^	15.2 ± 2.2 ^b^	< 0.01 *
**Heart rate recovery time** (seconds)	54.4 (36.2–111.5)	27.5 (22.0–51.5) ^b^	29.0 (20.2–35.1) ^b^	< 0.01 ^
**Respiratory rate recovery time** (seconds)	60.7 (35.8–101.3)	23.1 (15.7–30.5) ^b^	16.2 (11.1–20.3) ^b^	< 0.01 ^

Abbreviations: *FEV*_*1*_ Forced expiratory volume in 1 s, *FEF*_*25–75*_ Forced expiratory flow between 25 and 75% of the expiratory volume, *PEF* Peak expiratory flow, *n.o.u.* Number of use, *min* Minute, *h* Hour, *a.u.* Arbitrary unit

^a^*P* < =0.05 compared to controlled asthma

^b^*P* < =0.05 compared to uncontrolled asthma

*Normally distributed (ANOVA)

^ Not normally distributed and/or inhomogeneity of variances (Kruskal-Wallis)

### Multivariate analysis

**Fig. 3:**
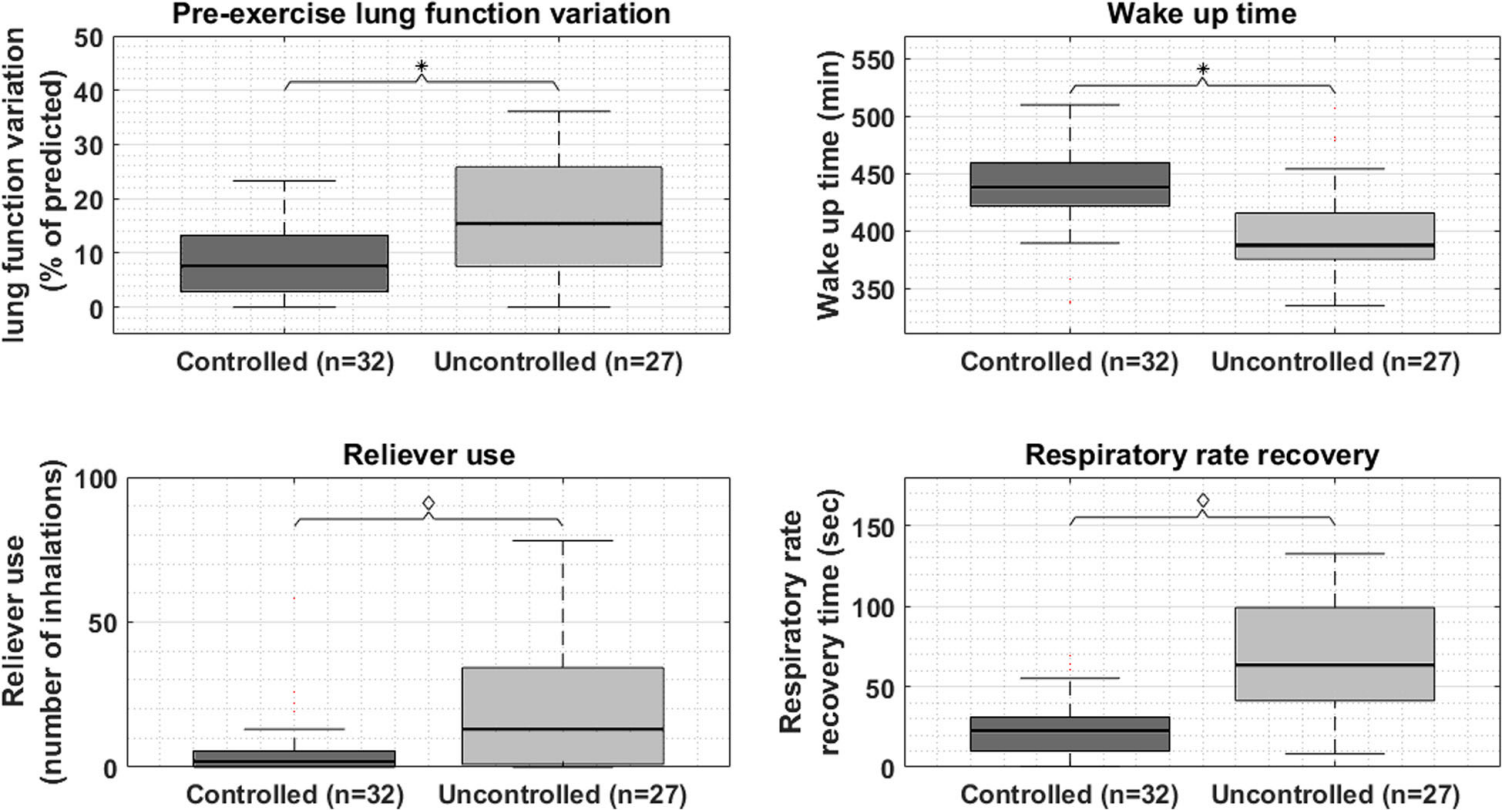
Distribution of the monitoring parameters. Legend: The boxplots (median, IQR and extreme values) display the distribution of the four significant contributors (pre-exercise lung function variation, wake-up-time, reliever use and respiratory rate recovery) to the multivariate binary logistic regression model after Markov Chain Monte Carlo imputation. The asterisks indicate significance with *p* < 0.05, the diamonds indicate significance with *p* < 0.01

11.5% Of missing data values were imputed using the Markov Chain Monte Carlo method, as missing patterns were random without monotonicity. Stepwise entering of the home-monitoring parameters for controlled and uncontrolled asthmatic children resulted in a multiple logistic regression model (*N* = 59) with R^2^ = 0.82. The final model (Table 3) showed that a larger variation in pre-exercise lung function (OR = 1.34 95%-CI 1.07–1.68), an earlier wake-up-time (OR = 1.05 95%-CI 1.01–1.10), more reliever use (OR = 1.11 95%-CI 1.03–1.19) and a longer respiratory rate recovery time (OR = 1.12 95%-CI 1.05–1.20) were associated with higher odds of being in the uncontrolled asthma group, compared to the controlled asthma group. Figure 3, display the distribution of the four significant contributors (pre-exercise lung function variation, wake-up-time, reliever use and respiratory rate recovery time) to the multivariate binary logistic regression model after Markov Chain Monte Carlo imputation. Table 4, shows the classification matrix of the model. Twenty-four of the twenty-seven uncontrolled asthmatic children (88.9% sensitivity) and 29 of the 32 controlled asthmatic children (90.6% specificity) can be accurately classified with the model. The associated positive and negative predicted values for uncontrolled and controlled asthma are 88.9 and 90.6%, respectively.

**Table 3:** The model characteristics of the binary logistic regression model

Covariates	Coefficient	***p***-value	Odds ratio	95% confidence interval	
				Lower	Upper
**Variation pre-exercise FEV**_**1**_	0.292	0.012	1.339	1.067	1.680
**Wake-up-time**	−0.053	0.012	0.948	0.910	0.988
**Reliever use**	0.100	0.006	1.105	1.029	1.187
**Respiratory rate recovery time**	0.113	0.001	1.120	1.046	1.198

**Table 4:** Classification matrix of the multivariate model: Paediatrician assessed control of asthma versus model based prediction of asthma control with multivariate binary logistic regression model

		Paediatrician based asthma control	
		Uncontrolled	Controlled
**Model based asthma control**	**Uncontrolled**	24	3
	**Controlled**	3	29

## Discussion

This study showed that data acquired from home-monitoring devices is strongly associated with the control of asthma, as assessed in the outpatient-clinic during an extensive evaluation including a bronchoprovocation test. The variation in lung function, the wake-up-time, the reliever usage and the recovery time of the respiratory rate after exercise did significantly distinguish between controlled and uncontrolled asthma in univariate analysis. Most striking is that the combination of these parameters can accurately identify 88.9% of all uncontrolled asthmatic children, suggesting a high potential of a holistic monitoring approach to assess paediatric asthma control at home.

To our knowledge, no studies are available using a multi-dimensional wearable monitoring approach in children with asthma to objectively assess asthma control, making WEARCON unique through its innovative approach of using state of the art technology. Honkoop et al. [38] published their study protocol about the prediction of exacerbations and deterioration in asthma control in adults using mHealth. Their approach resembles the WEARCON protocol in measuring spirometry, respiratory rate, physical activity and medication adherence.

Univariate analysis showed a significant difference in the variation in FEV_1_, which implies that uncontrolled asthmatic children show a wider range of pre-exercise FEV_1_ (mean 18.0%). Results of Brouwer et al. [39] are in line with our results. They found a mean FEV_1_ variation of 5.7% and suggested a disease cut-off of 11.8%. In their follow up research in 2010 Brouwer et al. [40] concluded that the contribution of FEV_1_ variation in diagnosing asthma in children is limited. Their study however aimed to differentiate asthmatic from non-asthmatic children, which may explain the different findings as controlled and uncontrolled asthmatic children were merged in one group.

The uncontrolled asthmatic children woke up earlier compared to the controlled asthmatic children. This is compatible with the circadian rhythms of asthma mediators such as cortisol and histamine [41]. Although previous studies found that children with uncontrolled asthma wake-up more often during night [42, 43], the wake-up-time was not previously found to be altered in children with uncontrolled asthma. Van Maanen et al. [44] found no differences in sleep parameters between children with frequent asthma symptoms and children without symptoms in the PIAMA birth cohort study, but no electronic sleep monitoring was used and they questioned whether their asthma questions on nocturnal asthma were sensitive enough to find an effect.

The GINA asthma strategy states that children with high use of short-acting bronchodilators are at risk for uncontrolled asthma [5]. The results of the WEARCON study correspond with that statement as the odds ratio indicates that every additional inhalation over a two-week period increases the risk of uncontrolled asthma with 10.5%. This emphasizes the importance of assessing inhaler use objectively with smart inhaler technology. Moreover, the reliever use data shows a high variability among children within the two asthma groups. We do believe that the classification of asthma control based on the amount of reliever use should therefore be made with caution, and in combination with other objective parameters, as poor symptom perception may influence the reliever use behavior.

The respiratory rate recovery time after exercise was on average almost twice as long (40 s) in children with uncontrolled asthma compared to children with controlled asthma. This seems small, but hampers children’s typical frequent short bust of intense activity [45]. No other studies investigated this parameter in asthmatic children. Post-exercise recovery in adolescents and adults is mediated by change in the RR and in the tidal volume. However, in children the RR recovery is the main contributor [46]. In children with uncontrolled asthma, the recovery of respiratory rate after exercise may be increased as bronchoconstriction compromises ventilation. Therefore, we expect the RR recovery to be a reproducible measure, just depending on the bronchoconstriction severity and possibly the cardio respiratory fitness. This is important to explore in a validity and reproducibility study.

Several single parameters could significantly distinguish between controlled and uncontrolled asthma in univariate analysis, which may reveal a suggestion for the individual patient whether his/her asthma is controlled or not. However, as Fig. 3 reveals, there is quite some overlap between the controlled and uncontrolled group, so the parameters in isolation may not provide sufficient accuracy, as previously found in literature [18–22]. This also holds true for the clinical practice as clinicians will not let them guide based on a single question/answer during a patient visit. Clinicians are trained to combine all the factors to come up with the right diagnosis. The multivariate model resembles this viewpoint and based on the results of this study do provide a more accurate classification of asthma control compared to the GINA questions alone.

A limitation of this study is that the non-asthmatic group was not matched to the asthma groups for gender. Prevalence of asthma is higher in boys than girls [1]. This corresponds with the baseline characteristics of the asthma groups in this study. However, our non-asthmatic group is 50/50 divided, possibly confounding univariate comparison between the asthmatic groups and the non-asthmatic children for several home-monitoring parameters (e.g. the amount of vigorous activities [47]). Nevertheless, the multivariate model was not affected by this limitation as the model was solely build on the data of the asthmatic children.

Although the results of this study emphasize the potential relevance of home-monitoring, further studies should validate the model of the WEARCON study. The model has been built on a training dataset of 60 asthmatic children, but has to be validated with a validation dataset of home-monitoring data in asthmatic children to determine the exact effect size.

The implication of the observations in our study is that a tool to reliably monitor asthma control at home seems attainable. Moreover, children were adherent to the home-measurements for the study period of 2 weeks. Children and parents embraced home-monitoring as was shown in the high participation rate and high adherency. However, for long-term asthma care the home-monitoring tool should be lean, non-obtrusive and proportional to the severity of the disease to maximize usability, engagement and minimize the burden to the child [48]. Such a tool could be a stepping stone to better follow the fluctuations of the asthma status and timely anticipate on signalled changes in asthma control. This could improve the current clinical evaluation of asthma control, which is intermittent and subjective. Proper randomized controlled trials and longitudinal studies will be needed to establish the efficacy of home-monitoring on asthma control when implemented in the paediatric asthma care.

## Conclusion

This study shows a correlation between data of home-monitoring devices and hospital-based assessment of asthma control. These results add to the rapidly expanding research field of home-monitoring of chronic respiratory diseases and provide a stepping stone to investigate paediatric asthma monitoring outside the hospital.

## Data Availability

The datasets generated and analysed during the current study are not publicly available, as the datasets contain secondary outcome parameters which will be used for follow up research in Medisch Spectrum Twente, but the datasets are available from the corresponding author on reasonable request.

## Abbreviations

(c-)ACT: (childhood) Asthma Control Test
ANOVA: Analysis of variance
ATS: American Thoracic Society
BPT: Broncho provocation test
CI: Confidence interval
ECG: Electrocardiography
ERS: European Respiratory Society
FEF2575: Forced Experatory Flow between 25 and 75% of expiratory volume
FEV1: Forced Expiratory Volume in 1 s
GINA: Global initiative for asthma
HR: Heart rate
IQR: Interquartile range
OR: Odds ratio
PEF: Peak Expiratory Flow
RR: Respiratory rate
SD: Standard deviation
WEARCON: Wearable home-monitoring of asthma control
